# ASO Author Reflections: R0 Resection After Oncologic Esophagectomy—Cutting on the Edge (0 cm) is Not Enough

**DOI:** 10.1245/s10434-021-10167-y

**Published:** 2021-05-21

**Authors:** Penelope St-Amour, Markus Schäfer, Styliani Mantziari

**Affiliations:** 1 Department of Visceral Surgery, University Hospital of Lausanne, Lausanne, Switzerland; 2 Faculty of Biology and Medicine, University of Lausanne (UNIL), Lausanne, Switzerland

## Past

The Union for International Cancer Control (UICC) tumor-node-metastasis (TNM) classification system has one outstanding characteristic: the individual definitions of the T, N, and M stages can be changed if novel evidence is occurring.^1^ Indeed, for many tumor entities, including esophageal cancer, these definitions have been adapted over time. Yet, the definition of the resection status (R) seemed to be carved in stone for a long time.

## Present

The R status bears crucial information on many levels. It shows the radicalness of surgery, the local tumor extension in addition to the T stage, the pattern of tumor growth, and the efficacy of neoadjuvant treatment. The R status is therefore of major prognostic value, together with other parameters.^2^ Nevertheless, two definitions currently are used (simultaneously) to describe a microscopically complete resection (R0). The American definition requires a direct contact (0 cm) with the tumor, whereas the Royal College suggests a larger margin (> 0.1 cm). Across published studies, it is not always clearly determined which of these definitions is used.

In the literature, wide variations exist in the reporting of R1 (and respectively R0) margins, according to the selected definition. Rates of R1 range from 15.3% with the American criteria (0 cm) to more than 36.5% with the Royal College criteria (< 0.1 cm).^3^ The authors’ results suggest higher sensitivity with the Royal College definition (> 0.1 cm) of negative margin status (R0) after oncologic esophagectomy.^4^ Significantly more adverse features (lymphovascular invasion, poor response to neoadjuvant treatment) are associated with microscopic resection margins smaller than 0.1 cm. This was reflected in similarly poor long-term survival for patients with 0- to 0.1-cm resection and those with 0-cm resection, although a lower recurrence risk was observed for the 0- to 0.1-cm patients.

## Future

The authors strongly believe that a well-accepted standardized definition of R0 is mandatory in the future to provide comparable and clinically meaningful results. The authors’ current study^3^ as well as previous studies^4^ suggest that although the 0-cm margin may define a higher-risk group, both the 0.0-cm and < 0.1-cm margins have a prognosis inferior to that of R0 and should be considered as R1.
